# Description of human anti-rabies post-exposure prophylaxis care notifications in Brazil, 2014-2019

**DOI:** 10.1590/S2237-96222022000200002

**Published:** 2022-06-15

**Authors:** Nathalie Mendes Estima, Marcelo Yoshito Wada, Silene Manrique Rocha, Deborah Sicchierolli Moraes, Patrícia Miyuki Ohara, Alexander Vargas, Dalva Maria de Assis

**Affiliations:** 1 Ministério da Saúde, Secretaria de Vigilância em Saúde, Brasília, DF, Brazil.

**Keywords:** Rabies, Post-Exposure Prophylaxis, Rabies Vaccines, Epidemiology, Descriptive, Public Health

## Abstract

**Objective:**

To analyze human anti-rabies post-exposure prophylaxis notifications in Brazil.

**Methods:**

This was a descriptive study using data from the Notifiable Diseases Information System in Brazil, from 2014 to 2019.

**Results:**

A total of 4,033,098 anti-rabies medical consultations were notified, averaging 672,183 a year. Percentage care was higher among males (n = 2,111,369; 52.4%), those under 19 years old (n = 1,423,433; 35.3%), living in urban areas (n = 3,386,589; 88.1%), attacked by dogs (n = 3,281,190; 81.5%) and bitten (n = 3,575,717; 81.9%), mainly on the hands and feet (n = 1,541,201; 35.3%). The most frequent prophylactic procedure was observation plus vaccination (n = 1,736,036; 44.2%). Prophylactic procedure was appropriate in 57.8% (n = 2,169,689) of cases and inappropriate in 42.2% (n = 1,582,411) of cases.

**Conclusion:**

Although there were appropriate prophylactic procedures, we also found procedures that were inappropriate and which, when insufficient, can result in cases of human rabies and, when unnecessary, can result in waste, including shortage of immunobiological products.

**Study contributions**

**Main results**

Anti-rabies care was provided, mainly, to males, those under 19 years old, living in urban areas, bitten by dogs on the hands and feet. In general prophylactic procedure was appropriate, with observation plus vaccination being most frequent.

**Implications for services**

Inappropriate or insufficient prophylactic procedures can lead to cases of human rabies and, when excessive, can lead to shortage of immunobiological products. Health professionals involved in indicating prophylaxis should receive updated information regularly.

**Perspectives**

Health authorities need to continue their efforts to prevent, control and eliminate rabies, seeking to achieve the World Health Organization goal for 2030: zero human dog-mediated rabies deaths.

## Introduction

Rabies is a communicable viral disease, characterized by acute progressive encephalitis, with a high case fatality ratio bordering 100%.^1^ This anthropozoonosis is transmitted to humans by the salivary secretions of infected mammals, mainly through bites on the hands or feet. Animals with the potential to transmit rabies are grouped into different transmission cycles, interrelated (to each other) and interacting with the human species: the urban cycle (dogs and cats), the airborne sylvatic cycle (bats), the terrestrial sylvatic cycle (foxes, primates, raccoons etc.) and the rural cycle (cattle, horses, goats and other animals of economic interest).^2^

Rabies is widely distributed around the world, with transmission in more than 150 countries.^3^ Human deaths are concentrated on the Asian and African continents, which together account for an estimated 56,000 deaths annually, mainly from attacks by domestic dogs infected with viruses of the *Lyssavirus* genus.^3^ Given the magnitude of the disease worldwide, the World Health Organization has set the goal of eliminating human rabies deaths due to canine variant rabies by the year 2030.^3^ In Brazil, 594 cases of human rabies were reported between the 1990s and 2017, mostly in urban areas and caused by the canine *Lyssavirus* variant.^4^

The various rabies prevention and control actions, which involve massive vaccination campaigns for dogs and cats, besides the blocking of animal hotspots and laboratory surveillance, have promoted a decrease in the occurrence of human cases, especially those related to transmission in the urban cycle. However, since 2000, there has been an increase in cases resulting from attacks by wild animals, especially chiroptera.^5^

The main form of human rabies prevention is post-exposure prophylaxis, which includes immediate washing of the wound with soap and water, observation of the attacking animal, usually dog or cat, for ten days and administration of immunobiologicals (vaccine and anti-rabies serum; or immunoglobulin), the indication of which depends on the type of exposure, the characteristics of the wound, the species and the status of the attacking animal (whether dog, cat or wild animal), according to the guidelines recommended by the Ministry of Health. 

In 2017, based on scientific evidence, the Ministry recommended changing the complete rabies post-exposure prophylaxis regimen, namely reducing treatment from 5 to 4 vaccine doses.^2^ ^,^ ^5^ ^-^ ^7^ An economic evaluation study on federal expenditure by the National Immunization Program between 2004 and 2015 (the period before the change in the prophylactic regimen) showed that over the 12-year period the Brazilian National Health System (SUS) invested approximately BRL 821 million purchasing immunobiologicals for human rabies prophylaxis.^4^

On average 591,871 notifications were recorded annually in Brazil between 2009 and 2013.^5^ Considering its great relevance for public health, due to the high case fatality ratio and high costs related to prophylaxis and health care, any accident involving an animal with the potential to transmit rabies must obligatorily be notified immediately, at the municipal level of the health system. Cases are registered by filling out and inputting an ‘Investigation Form - Human Anti-rabies Consultation’ on the Notifiable Health Conditions Information System (SINAN), regardless of whether or not the injured person is diagnosed as needing prophylaxis.^2^ ^,^ ^8^ ^,^ ^9^

The processing, analysis and dissemination of data on human anti-rabies post-exposure prophylaxis contribute to the monitoring, planning, evaluation and improvement of both health surveillance actions and health services themselves. 

The objective of this study was to analyze human anti-rabies post-exposure prophylaxis notifications in Brazil between 2014 and 2019.

## Methods

This was a descriptive study of human anti-rabies post-exposure prophylaxis care notified on the SINAN system in Brazil between 2014 and 2019.

We analyzed data on accidents caused by any animal with the potential to transmit rabies notified by health services. Animals with the potential to transmit rabies are considered to be mammals in general: dogs, cats, wild animals (chiroptera, foxes, crab-eating foxes, Geoffroy’s cats, striped hog-nosed skunks, South American raccoons, marsupials, primates), cattle and horses, among others.^2^

The variables of interest analyzed were:

- Sociodemographic
  - Sex (male; female);
  - Age group (in years: under 1; 1-19; 20-39; 40-59; 60 and over);
  - Race/skin color (Brown; White; Black; Yellow; Indigenous);
  - Schooling (illiterate; elementary education I; elementary education II; high school education; higher education);
  - Zone of residence (urban; rural; peri-urban); and
  - Federative Unit of residence.
- Epidemiological background
  - Type of exposure (bite; scratch; lick; indirect contact; other);
  - Location of the wound (hands and feet; lower limbs; upper limbs; head/neck; torso; mucous membrane);
  - Wound (single; multiple; not wounded);
  - Type of wound (surface; deep; laceration);
  - Attacking animal species (dog; cat; bat; primate; fox; domestic herbivore; other); and
  - Status of the animal, for the purposes of prophylactic procedure (healthy; suspect; dead/missing; rabid).
- Current prophylaxis
  - Final status of the animal [rabies negative (clinical); rabies negative (laboratory); dead/put down/no diagnosis; rabies positive (clinical) and rabies positive (laboratory)];
  - Prophylaxis indicated (prophylaxis waived; observation of animal; observation plus vaccination; vaccination; anti-rabies serum plus vaccination; reexposure regimen);
  - Prophylaxis interrupted (yes; no);
  - Reason for interruption (dropout; indicated by health center; transfer);
  - Active tracing, when there was prophylaxis dropout and the health center went in search of the wounded person (yes; no); and
  - Anti-rabies serum indicated (yes; no).

In order to identify the prophylactic procedure indicated, which we defined as appropriate or inappropriate, human anti-rabies post-exposure prophylaxis was first classified according to exposure type, as per Ministry of Health guidelines: indirect contact; accidents involving wild animals; severe accidents; and minor accidents.^2^ Accidents were classified as severe when i) the wound was located in mucous membrane, the head and neck, or the hands and feet, ii) the wound was multiple, or iii) the wound was deep or lacerated. Other accidents that did not meet this definition were classified as minor. Indirect contact refers to accidents that involve handling potentially contaminated utensils, for example. 

In the case of prophylactic procedures initially classified as inappropriate, a further sub-classification was established, as either ‘insufficient’ or ‘excessive’ procedures, suggesting that the prophylaxis indicated was not in accordance with health authority recommendations, because it was either insufficient or excessive. Prophylactic procedure classified as appropriate suggests that the prophylaxis indicated followed the national norms.^6^ ^,^ ^10^ ^,^ ^11^

The data on human rabies care were taken from the SINAN database. The system is managed by the Health Ministry’s Department of Health Analysis and Noncommunicable Disease Surveillance. The data were extracted on June 24, 2020. 

We performed descriptive analysis of the data, calculating absolute and relative frequencies, measures of central tendency and dispersion (mean and standard deviation), and incidence rates. Incidence rates were obtained by taking the ratio between the absolute number of notifications by Federative Unit of residence in the years 2014-2019 and the population estimated for 2017 by the Brazilian Institute of Geography and Statistics, multiplied by 1,000.^12^ Variables with more than 50% unknown or blank records were excluded from the analysis (considering this completeness parameter, which ranges from low to very low).^13^ ^,^ ^14^ We used Excel 2013® to process and analyze the data.

The research project was submitted to the National Health Council’s National Research Ethics Committee on November 13, 2020, and approved as per Opinion No. 4.396.733 - Certificate of Submission for Ethical Appraisal No. 39003820.9.0000.0008 -, in accordance with National Health Council Resolution No. 466, dated December 12, 2012.

## Results

A total of 4,128,364 anti-rabies medical consultations were notified in Brazil between 2014 and 2019, of which 97.7% (n = 4,033,098) were post-exposure prophylaxis and 2.3% (n = 95,266) were pre-exposure prophylaxis. The latter were excluded from the analysis. On average there were 672,183 (DP: ±41.238.3) post-exposure prophylaxis notifications per year. There was an 11% increase in the number of notifications between 2014 (n = 645,335) and 2019 (n = 716,455).

Regarding the sociodemographic profile of the people receiving care, most were male (n = 2,111,369; 52.4%), 1 to 19 years old (n = 1,368,486; 33.9%), of Brown skin color (n = 1,604,912; 46.5%) or White skin color (n = 1,575,682; 45.7%), and had level II elementary education (n = 711,513; 34.5%). Most human anti-rabies post-exposure prophylaxis occurred in urban areas (n = 3,386,589; 88.1%) (Table 1).

**Table 1:** Distribution of human anti-rabies post-exposure care (n = 4,033,098) according to sociodemographic characteristics, Brazil, 2014-2019

Sociodemographic characteristics	n	%
**Sex (n = 4,031,640)**		
Male	2,111,369	52.4
Female	1,920,271	47.6
**Age group (in years) (n = 4,033,084)**		
<1	54,947	1.4
1-19	1,368,486	33.9
20-39	1,054,952	26.2
40-59	947,557	23.5
≥60	607,142	15.0
**Race/skin color (n = 3,451,110)**		
Brown	1,604,912	46.5
White	1,575,682	45.7
Black	224,721	6.5
Yellow	28,436	0.8
Indigenous	17,359	0.5
**Schooling (n = 2,063,631)^a^**		
Illiterate	66,629	3.2
Elementary education I	562,932	27.3
Elementary education II	711,513	34.5
High school education	555,174	26.9
Higher education	167,383	8.1
**Zone of residence (n = 3,842,981)**		
Urban	3,386,589	88.1
Rural	436,408	11.4
Peri-urban	19,984	0.5

a) Does not apply to under-7-year-olds (n = 557,923).

**Figure 1:**
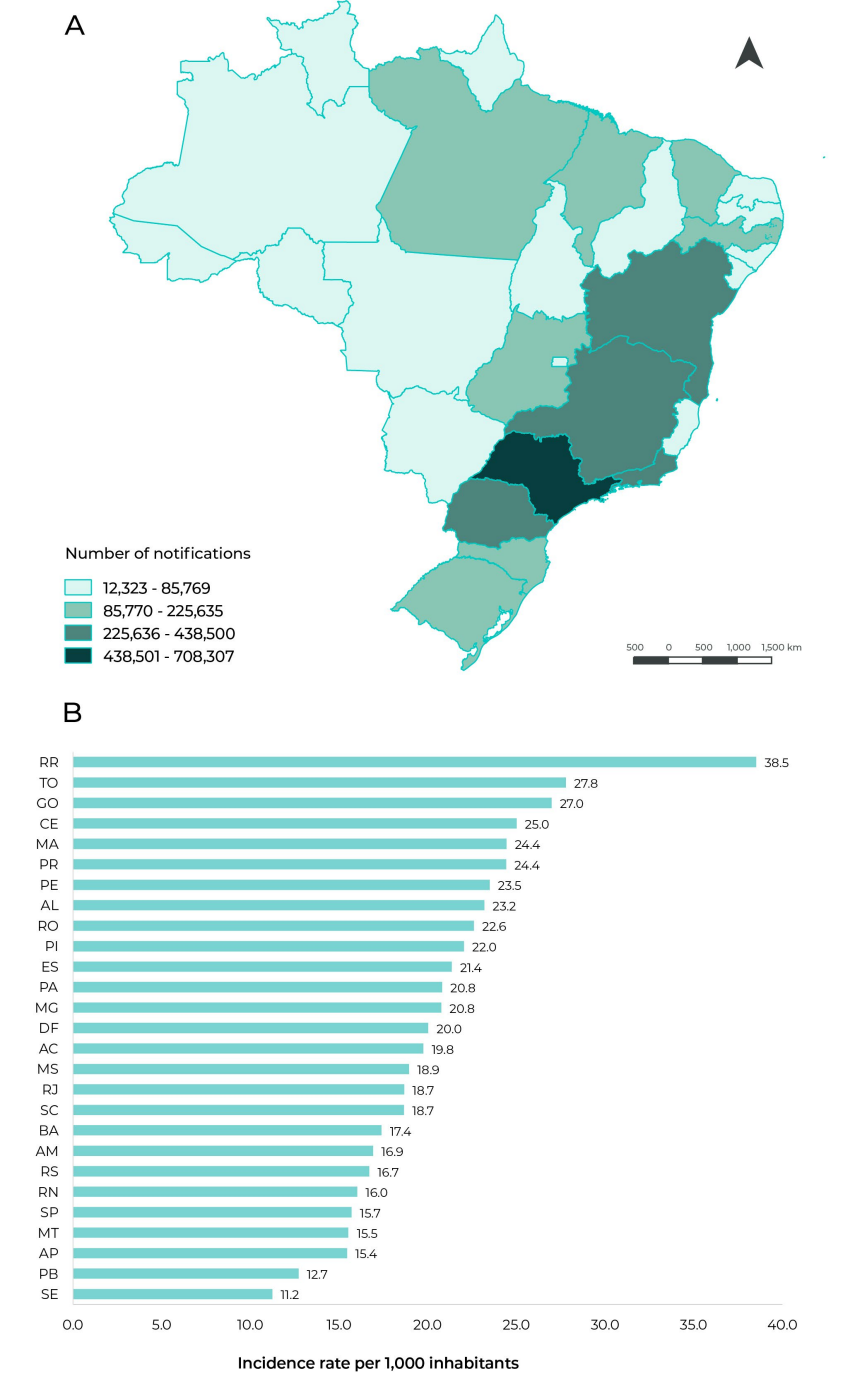
Distribution of human anti-rabies post-exposure care (n = 4,033,098) according to Federative Unit of notification (A) and incidence rate per 1,000 inhabitants (B), Brazil, 2014-2019

The states with the most human anti-rabies post-exposure prophylaxis notifications were São Paulo, with 708,307 (17.6%), followed by Minas Gerais, with 438,500 (10.9%), and Rio de Janeiro, with 312,107 (7.7%). Amapá, Acre and Roraima with 12,323 (0.3%), 16,387 (0.4%) and 20,134 (0.5%) notifications, respectively, had the lowest frequency of reported cases. With regard to incidence rates, obtained based on recorded notifications, the states of Roraima and Tocantins had the highest rates, *i.e.* 38.5 and 27.8 per 1,000 inhabitants, respectively (Figure 1).

Regarding the variables related to epidemiological background, bites and scratches together accounted for 95.6% (n = 4,176,973) of the records. The hands and feet comprised 35.3% (n = 1,541,201) of injury sites, followed by the lower limbs 34.1% (n = 1,488,110); wounded mucous membranes accounted for 2.1% (n = 89,807) of notifications. Single wounds accounted for 59.8% (n = 2,336,416) of the cases, 51.0% (n = 2,015,477) were surface wounds, while 43.6% were deep wounds (n = 1,719,434) (Table 2).

**Table 2:** Distribution of human anti-rabies post-exposure care (n = 4,033,098) according to epidemiological background and current prophylaxis, Brazil, 2014-2019

Variables	n	%
**Epidemiological background**		
**Type of exposure^a^ (n = 4,368,254)**		
Bite	3,575,717	81.9
Scratch	601,256	13.7
Lick	105,643	2.4
Indirect contact	52,294	1.2
Other	33,344	0.8
**Location of the wound^a^ (n = 4,360,995)**		
Hands/feet	1,541,201	35.3
Lower limbs	1,488,110	34.1
Upper limbs	704,660	16.2
Head/neck	337,007	7.7
Torso	200,210	4.6
Mucous membrane	89,807	2.1
**Wound (n = 3,908,181)**		
Single	2,336,416	59.8
Multiple	1,541,099	39.4
Not wounded	30,666	0.8
**Type of wound^a^ (n = 3,948,063)**		
Surface	2,015,477	51.0
Deep	1,719,434	43.6
Laceration	213,152	5.4
**Attacking animal species (n = 4,028,237)**		
Dog	3,281,190	81.5
Cat	612,690	15.2
Bat	31,786	0.8
Primate	19,769	0.5
Fox	5,661	0.1
Domestic herbivore	11,288	0.3
Other	65,853	1.6
**Status of the animal (n = 3,872,508)**		
Healthy	2,762,232	71.3
Suspect	638,614	16.5
Dead/missing	455,362	11.8
Rabid	16,300	0.4
**Current prophylaxis**		
**Final status of the animal (n = 2,420,706)**		
Rabies negative (clinical)	2,083,834	86.1
Dead/put down/no diagnosis	283,273	11.7
Rabies negative (laboratory)	42,593	1.8
Rabies positive (clinical)	5,057	0.2
Rabies positive (laboratory)	5,949	0.2
**Prophylaxis indicated (n = 3,929,787)**		
Observation + vaccination	1,736,036	44.2
Observation of animal	1,044,030	26.6
Vaccination	754,452	19.2
Anti-rabies serum + vaccination	329,124	8.4
Prophylaxis waived	56,850	1.4
Reexposure regimen	9,295	0.2
**Prophylaxis interrupted (n = 2,226,105)**		
No	1,684,981	75.7
Yes	541,124	24.3
**Reason for interruption (n = 541,124)**		
Dropout	339,356	62.7
Indicated by health center	167,363	30.9
Transfer	34,405	6.4
**Active tracing, carried out by health center (n = 307,048)**		
Yes	236,862	77.1
No	70,186	22.9
**Anti-rabies serum indicated (n = 2,133,632)**		
No	1,867,673	87.5
Yes	265,959	12.5

a) More than one category allowed.

Regarding the species of the attacking animals, 96.7% (n = 3,893,880) corresponded to attacks related to the urban cycle of rabies transmission, that is, by dogs and cats. Wild animals, such as chiropterans, primates and foxes, accounted for 1.4% (n = 57,216) of the records. Just 1.6% (n = 65,853) of the notifications involved other animal species, and in these cases, accidents were recorded involving both animals that potentially transmit the disease (other mammals) and animals that are not part of the transmission cycle (arthropods, amphibians, reptiles and birds). Healthy animals accounted for 71.3% (n = 2,762,232) of total notifications (Table 2).

As for the variables related to current prophylaxis, the final status of the animal, when taking the clinical and laboratory criteria together, was rabies negative in 87.9% (n = 2,126,427) of the cases. Indication of prophylaxis comprising observation plus vaccination accounted for 44.2% (n = 1,736,036) of the cases, while vaccination-only prophylaxis accounted for 19.2% (n = 754,452), and prophylaxis with anti-rabies serum plus vaccination accounted for 8.4% (n = 329,124). Prophylaxis was interrupted in 24.3% (n = 541,124) of the cases, mostly due to dropout: 62.7% (n = 339,356). Active tracing was performed by health centers in relation to 77.1% (n = 236,862) of those who interrupted prophylaxis due to dropout. Prophylaxis with anti-rabies serum was indicated in 12.5% (n = 265,959) of all notifications (Table 2).

**Figure 2:**
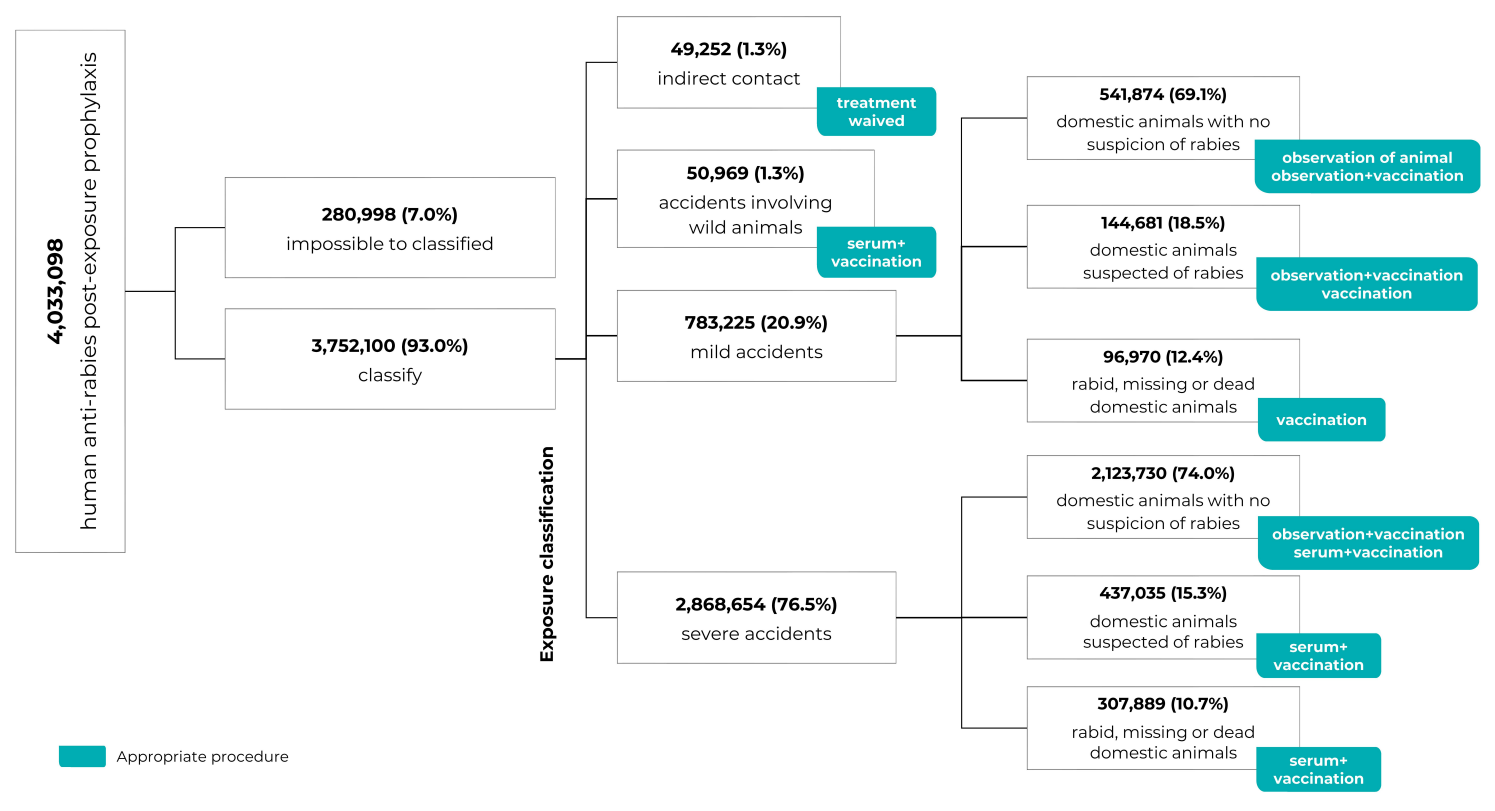
Flowchart of classification of human anti-rabies post-exposure prophylaxis medical consultations (n = 4,033,098) according to exposure classification and appropriate prophylactic procedure, Brazil, 2014-2019

Note: Appropriate procedures were classified as follows: indirect contact (treatment waived); accidents involving wild animals (anti-rabies serum + vaccination); minor accidents involving domestic animals not suspected of having rabies (observation of the animal or observation + vaccination); minor accidents involving domestic animals suspected of having rabies (observation + vaccination or vaccination); minor accidents involving rabid, missing or dead domestic animals (vaccination); severe accidents involving domestic animals not suspected of having rabies (observation + vaccination or anti-rabies serum + vaccination); severe accidents involving domestic animals suspected of having rabies (anti-rabies serum + vaccination); and severe accidents involving rabid, missing or dead domestic animals (anti-rabies serum + vaccination).

With regard to exposure type and the prophylactic procedure used in human anti-rabies post-exposure prophylaxis, we were able to classify 93.0% (n = 3,752,100) of the notifications; 7.0% (n = 280,998) were not classified because they had incomplete data. Among the records that were classified, 1.3% (n = 49,252) were indirect contact, 1.3% (n = 50,969) were accidents involving wild animals, 20.9% (n = 783,225) were classified as minor accidents, and 76.5% (n = 2,868,654) as severe accidents (Figure 2).

Regarding the appropriateness of the pro-phylactic procedure indicated, among exposures classified as indirect contact (n = 49,252), 3.3% (n = 1,648) were classified as appropriate (prophylaxis waived). In the case of minor accidents involving domestic animals not suspected of having rabies (n = 541,874), 85.9% (n = 465,891) of the prophylactic procedures - observation of the animal; observation plus vaccination - were appropriate. Among minor accidents involving domestic animals suspected of rabies (n = 144,681), appropriate prophylactic procedures (observation plus vaccination; vaccination) accounted for 77.8% (n = 112,505) of notifications, while for minor accidents involving rabid, missing or dead domestic animals (n = 96,670), appropriate prophylactic procedures (vaccination) accounted for 81.4% (n = 78,663) (Table 3).

**Table 3:** Distribution of human anti-rabies post-exposure care (n = 3,752,100) according to prophylactic procedure indicated, Brazil, 2014-2019

Prophylactic procedure	Appropriate		Inappropriate			
			Insufficient		Excessive	
	n	%	n	%	n	%
**Indirect contact (n = 49,252)**						
Prophylaxis waived	1,648	3.3	-^a^	-^a^	-^a^	-^a^
Observation of animal	-^a^	-^a^	-^a^	-^a^	8,929	18.1
Observation + vaccination	-^a^	-^a^	-^a^	-^a^	17,468	35.5
Vaccination	-^a^	-^a^	-^a^	-^a^	13,656	27.7
Anti-rabies serum + vaccination	-^a^	-^a^	-^a^	-^a^	7,551	15.3
**Minor accidents with domestic animals not suspected of having rabies (n = 541,874)**						
Prophylaxis waived	-^a^	-^a^	12,878	2.4	-^a^	-^a^
Observation of animal	294,670	54.4	-^a^	-^a^	-^a^	-^a^
Observation + vaccination	171,221	31.5	-^a^	-^a^	-^a^	-^a^
Vaccination	-^a^	-^a^	-^a^	-^a^	58,979	10.9
Anti-rabies serum + vaccination	-^a^	-^a^	-^a^	-^a^	4,126	0.8
**Minor accidents with domestic animals suspected of having rabies (n = 144,681)**						
Prophylaxis waived	-^a^	-^a^	2,361	1.6	-^a^	-^a^
Observation of animal	-^a^	-^a^	20,162	13.9	-^a^	-^a^
Observation + vaccination	45,193	31.3	-^a^	-^a^	-^a^	-^a^
Vaccination	67,312	46.5	-^a^	-^a^	-^a^	-^a^
Anti-rabies serum + vaccination	-^a^	-^a^	-^a^	-^a^	9,653	6.7
**Minor accidents with rabid, missing or dead domestic animals (n = 96,670)**						
Prophylaxis waived	-^a^	-^a^	2,051	2.1	-^a^	-^a^
Observation of animal	-^a^	-^a^	4,648	4.8	-^a^	-^a^
Observation + vaccination	-^a^	-^a^	1,091	1.1	-^a^	-^a^
Vaccination	78,663	81.4	-^a^	-^a^	-^a^	-^a^
Anti-rabies serum + vaccination	-^a^	-^a^	-^a^	-^a^	10,217	10.6
**Severe accidents with domestic animals not suspected of having rabies (n = 2,123,730)**						
Prophylaxis waived	-^a^	-^a^	20,137	0.9	-^a^	-^a^
Observation of animal	-^a^	-^a^	654,262	30.8	-^a^	-^a^
Observation + vaccination	1,247,007	58.7	-^a^	-^a^	-^a^	-^a^
Vaccination	-^a^	-^a^	167,116	7.9	-^a^	-^a^
Anti-rabies serum + vaccination	35,208	1.7	-^a^	-^a^	-^a^	-^a^
**Severe accidents with domestic animals suspected of having rabies (n = 437,035)**						
Prophylaxis waived	-^a^	-^a^	3,548	0.8	-^a^	-^a^
Observation of animal	-^a^	-^a^	43,361	9.9	-^a^	-^a^
Observation + vaccination	-^a^	-^a^	178,398	40.8	-^a^	-^a^
Vaccination	-^a^	-^a^	153,763	35.2	-^a^	-^a^
Anti-rabies serum + vaccination	57,965	13.3	-^a^	-^a^	-^a^	-^a^
**Severe accidents with rabid, missing or dead domestic animals (n = 307,889)**						
Prophylaxis waived	-^a^	-^a^	4,530	1.5	-^a^	-^a^
Observation of animal	-^a^	-^a^	2,523	0.8	-^a^	-^a^
Observation + vaccination	-^a^	-^a^	16,331	5.3	-^a^	-^a^
Vaccination	-^a^	-^a^	148,904	48.4	-^a^	-^a^
Anti-rabies serum + vaccination	135,601	44.0	-^a^	-^a^	-^a^	-^a^
**Accidents involving wild animals (n = 50,969)**						
Prophylaxis waived	-^a^	-^a^	803	1.6	-^a^	-^a^
Observation of animal	-^a^	-^a^	580	1.1	-^a^	-^a^
Observation + vaccination	-^a^	-^a^	3,607	7.1	-^a^	-^a^
Vaccination	-^a^	-^a^	10,778	21.1	-^a^	-^a^
Anti-rabies serum + vaccination	35,201	69.1	-^a^	-^a^	-^a^	-^a^
**Indirect contact (n = 49,252)**	1,648	3.3	-^a^	-^a^	47,604	96.7
**Minor accidents (n = 783,225)**	657,059	83.9	43,191	5.5	82,975	10.6
**Severe accidents (n = 2,868,654)**	1,475,781	51.4	1,392,873	48.6	-^a^	-^a^
**Accidents involving wild animals (n = 50,969)**	35,201	69.1	15,768	30.9	-^a^	-^a^
**Total**	**2,169,689**	**57.8**	**1,582,411**		**42,2**	

a) This type of classification is not possible.

Among the severe accidents involving domestic animals not suspected of rabies (n = 2,123,730), 60.4% (n = 1,282,215) of prophylactic procedures were appropriate (observation plus vaccination; anti-rabies serum plus vaccination). In the case of severe accidents involving domestic animals suspected of rabies (n = 437,035) and rabid, missing, or dead domestic animals (n = 307,889), procedures were inappropriate or insufficient (prophylaxis waiver; observation of the animal; observation plus vaccination; vaccination only) in 86.7% (n = 379,070) and 56.0% (n = 172,288) of cases, respectively. Regarding accidents involving wild animals (n = 50,969), prophylactic procedure was appropriate (anti-rabies serum plus vaccination) in 69.1% (n = 35,201) of the cases treated (Table 3).

Of the total human anti-rabies post-exposure prophylaxis care classified with regard to prophylactic procedure (n = 3,752,100), 57.8% (n = 2,169,689) of procedures were classified as appropriate and 42.2% (n = 1,582,411) as inappropriate. Among the inappropriate prophylactic procedures, 91.7% (n = 1,541,832) were considered insufficient, while 8.3% (n = 130,579) were considered excessive. Indirect contact and severe accidents corresponded to 36.5% (n = 47,604) of excessive procedures and 95.9% (n = 1,392,873) of insufficient procedures, respectively (Table 3).

## Discussion

During the period analyzed, more than 4 million notifications of human anti-rabies post-exposure prophylaxis were recorded, with highest absolute frequency of records in the Southeast region and the highest incidence rate in the Northern region of the country. Attacks occurred mostly among young males, with injuries to the hands and feet, caused by dogs and cats. Although prophylactic procedure was appropriately indicated in most cases of human anti-rabies post-exposure prophylaxis, nevertheless inappropriate indications for rabies prophylaxis were also made.

The high number of notifications suggests that both the population and the health care and surveillance community recognize the need to seek health care when suffering an attack by an animal with the potential to transmit rabies, and the importance of reporting this event for public health. 

However, it is important to emphasize that, because of the high case fatality ratio, seeking post-exposure prophylaxis is necessary, even for mild accidents, especially those caused by wild animals; even in situations where bats enter buildings, where the risk of exposure is unknown.^2^ However slight the attack, people should be advised to seek health care for assessment as to post-exposure prophylaxis, thus avoiding cases and sporadic outbreaks of human rabies caused mainly by bats. 

As the surveillance system is passive, it is likely that human anti-rabies post-exposure prophylaxis is not effectively captured by the information system, leading to underreporting in Brazil,^15^ especially in hard-to-reach rural areas, such as riverside areas in the Amazon, where cultural factors can also influence the occurrence of attacks, given the frequent contact with animals, including wild animals, and limited access to health services.^16^

An increase in the number of records was observed, especially when comparing the average number of notifications per year, between the period covered by this study and the five-year period previously analyzed (2009-2013), even though the same database was used in both studies.^5^ This increase may be related to the Ministry of Health issuing Information Note No. 26-SEI/2017, on the changes in the post-exposure prophylaxis regimen for human rabies, which may have made health professionals aware of the importance of human rabies prevention and control measures.^6^ Other factors, related to increased interactions between humans and animals, deforestation and unplanned urbanization of cities, for example, may also have contributed to the increase found.

Furthermore, as a result of the increase in notifications, it is possible that there will be an impact on the costs of SUS investments in human anti-rabies post-exposure prophylaxis actions, considering implications such as increased use of immunobiologicals, working time spent and human resources available for human rabies surveillance and care. In this sense, it is necessary to reflect on the importance of the indication of prophylactic procedure with emphasis on observation of dogs and cats for ten days, without immediate administration of immunobiologicals, when possible, as recommended by the World Health Organization.^3^ Considering the Brazilian epidemiological scenario, in which the last case of human rabies caused by the canine variant was recorded in 2015, this reflection is timely for the rational use of immunobiologicals and cost reduction for the SUS.^4^

The male population is more exposed to accidents involving animals with the potential to transmit rabies, possibly related to work activities.^1^ Furthermore, according to the evidence presented in a previous national analysis of human anti-rabies post-exposure prophylaxis records for the period 2009 to 2013, the most affected age group comprised young people (1 to 19 years old).^5^

The Southeastern region, represented by the states of São Paulo, Minas Gerais and Rio de Janeiro, in that order, concentrated the largest absolute numbers of notifications in the period under analysis, corroborating the results of previous studies, since the year 2000.^5^ ^,^ ^17^ However, the highest incidence rates of human anti-rabies post-exposure prophylaxis were found in the Northern region of the country, in the states of Roraima and Tocantins.

Dogs and cats, which comprise the urban rabies transmission cycle, are the attacking animal species most frequently associated with human anti-rabies post-exposure prophylaxis.^1^ ^,^ ^5^ ^,^ ^18^ However, it is important to note that the most recent records of human rabies in Brazil relate to attacks by wild animals, mainly bats.^4^ ^,^ ^19^ This points to a transition in human rabies transmission from the urban to the sylvatic cycle, especially the airborne cycle, with outbreaks reported not only in Brazil but also in other Latin American countries, such as Ecuador and Peru.^4^ ^,^ ^20^ ^,^ ^21^ Given the high case fatality ratio, seeking post-exposure prophylaxis is necessary, even in the case of mild wounds, especially those caused by wild animals.

This transition in the human rabies epidemiological scenario justifies the efforts of health authorities to eliminate transmission of the disease by dogs in Latin America and the Caribbean, primarily through mass dog vaccination campaigns.^22^ Special attention should be paid to secondary cases, due to infection of dogs and cats by bat variants, and to developing specific prevention and control strategies in this regard.^4^

Bites were the most common type of exposure among the notifications, and this can be explained by the fact that for aggressive animals biting is a way of defending themselves.^5^ The most affected sites were the hands and feet, since they are more frequently used as a form of protection against attacks, corroborating the findings of other authors.^17^ ^,^ ^18^ ^,^ ^23^ ^,^ ^24^ Moreover, the site and type of the wound and the status of the animal at the time of the attack are important for classifying accidents as mild or severe, considering the action of the virus on the central nervous system, in order to guide post-exposure prophylaxis procedures.^6^ ^,^ ^23^

The most indicated type of prophylaxis was observation plus vaccination, in keeping with the kind of attacking animal, mainly dogs and cats found to be negative for rabies according to clinical criteria.^5^ It is important to reflect on the possibility of making observation - without starting the prophylactic regimen using vaccine straightaway - the ideal procedure in the face of aggression by animals without clinical signs suggestive of rabies, and the possibility of their observation, in order to contribute to the rational use of immunobiologicals in health services, as recommended by the World Health Organization.^7^ We found that interruption of prophylaxis was mostly due to prophylactic therapy dropout, as described in other studies.^5^ ^,^ ^24^ In these cases, active tracing by health services to ensure completion of prophylaxis is essential. Failure to start or complete the appropriate prophylaxis regimen may result in cases of human rabies.

In general, prophylactic procedure was found to be appropriate for the human anti-rabies post-exposure prophylaxis cases. However, in the case of certain exposure types, such as indirect contact and involvement of domestic animals suspected of rabies and missing or dead rabid animals, a considerable number of human anti-rabies post-exposure prophylaxis were inappropriately conducted, either excessively or insufficiently. 

It is important to emphasize that inappropriate prophylactic procedure may lead to cases of human rabies if the prophylactic regimen involving administration of immunobiologicals (anti-rabies serum and vaccine) is insufficient, while its unnecessary (excessive) indication may cause waste and even result in shortages due to lack of immunobiologicals, besides exposing people to the risk of unnecessary adverse events.^9^ ^,^ ^10^ As such, constant receipt of updated information by health professionals involved in indicating post-exposure prophylaxis reinforcing the importance of washing the wound with soap and water, immediately after the attack, as well as adequate administration of immunobiologicals are fundamental.It is possible that the results of this study may have been influenced by certain limitations, arising from its very nature. The use of a secondary database may imply information bias in the case of incomplete and/or inconsistent notifications. Some notifications, for example, were not able to be classified as to severe or mild exposure type, so that the results of the study may be underestimated.

In view of the scenario presented, it is important that health authorities remain focused on rabies prevention, control and elimination in order to achieve the World Health Organization’s goal for 2030: zero human dog-mediated rabies deaths.

## References

[1] Wada MY, Rocha SM, Maia-Elkhoury ANS (2011). Situação da raiva no Brasil, 2000 a 2009. Epidemiol Serv Saude.

[2] Ministério da Saúde (BR) (2019). Guia de vigilância em saúde: volume único.

[3] Word Health Organization (2018). WHO expert consultation on rabies: third report.

[4] Vargas A, Romano APM, Merchán-Hamann E (2019). Raiva humana no Brasil: estudo descritivo,
2000-2017. Epidemiol e Serv saude.

[5] Ministério da Saúde (BR). Secretaria de Vigilância em Saúde.
Departamento de Vigilância Epidemiológica (2016). Perfil dos atendimentos antirrábicos humanos, Brasil,
2009-2013. Bol Epidemiológico.

[6] Ministério da Saúde (BR). Secretaria de Vigilância em Saúde.
Departamento de Vigilância das Doenças Transmissíveis (2017). Nota Informativa nº 26-SEI/2017-CGPNI/DEVIT/SVS/MS. Informa sobre
alterações no esquema de vacinação da raiva humana pós-exposição e dá outras
orientações. Protocolo Raiva 2017.

[7] World Health Organization (2018). Rabies vaccines and immunoglobulins: WHO position April 2018
[Internet].

[8] Ministério da Saúde (BR) (2020). Lista nacional de notificação compulsória de doenças, agravos e eventos
de saúde pública.

[9] Cavalcante KKS, Alencar CH (2018). Raiva humana: avaliação da prevalência das condutas profiláticas
pós-exposição no Ceará, Brasil, 2007-2015. Epidemiol e Serv saude.

[10] Ministério da Saúde (BR). Secretaria de Vigilância em Saúde (2019). Situação da raiva no Brasil e recomendações quanto ao uso dos
imunobiológicos. Bol Epidemiológico.

[11] Araujo IL (2017). Avaliação da profilaxia inicial pós-exposição da raiva humana , indicada
em acidentes notificados com gatos, em Belo Horizonte/MG , no período de
2007 a 2016 [dissertação].

[12] Instituto Brasieliro de Geografia e Estatística (2020). Estimativas da população [Internet].

[13] Ministério da Saúde (BR). Secretaria de Vigilância em Saúde.
Departamento de Vigilância das Doenças Transmissíveis. Coordenação-Geral
de Vigilância e Resposta às Emergências em Saúde Pública. Unidade
Técnica de Gestão do Sinan (2015). SINAN Relatórios - Manual de Operação [Internet].

[14] Abath MB, Lima MLLT, Lima PS, Silva MCM, Lima MLC (2014). Avaliação da completitude, da consistência e da duplicidade de
registros de violências do Sinan em Recife, Pernambuco,
2009-2012. Epidemiol Serv Saude.

[15] Organização Pan-Americana da Saúde (2010). Módulo de princípios de epidemiologia para o controle de enfermidades
(MOPECE): vigilância em saúde pública [Internet].

[16] Cavalcante KKS, Florêncio CMGD, Alencar CH (2019). Atendimentos antirrábicos humanos pós-exposição: tendência
temporal de sua prevalência no Ceará, de 2007 a 2015. Cad Saude Colet.

[17] Silva GM, Brandespim DF, Rocha MDG, Leite RMB, Oliveira JMB (2013). Notificações de atendimento antirrábico humano na população do
município de Garanhuns, estado de Pernambuco, Brasil, no período de 2007 a
2010. Epidemiol Serv Saude.

[18] Filgueira AC, Cardoso MD, Ferreira LOC (2011). Profilaxia antirrábica humana: uma análise exploratória dos
atendimentos ocorridos em Salgueiro-PE, no ano de 2007. Epidemiol Serv Saude.

[19] Ministério da Saúde (BR). Secretaria de Vigilância em Saúde
Departamento de Imunização e Doenças Transmissíveis (2020). Raiva humana por animais silvestres no Brasil: atualizações e
condutas profiláticas. Bol Epidemiológico.

[20] Ortiz-Prado E, Ponce-Zea J, Ramirez D, Stewart-Ibarra AM, Armijos L, Yockteng J (2016). Rabies epidemiology and control in Ecuador. Glob J Health Sci.

[21] Salmón-Mulanovich G, Vásquez A, Albújar C, Guevara C, Laguna-Torres VA, Salazar M (2009). Human rabies and rabies in vampire and nonvampire bat species,
southeastern Peru, 2007. Emerg Infect Dis.

[22] Vigilato MAN, Clavijo A, Knobl T, Silva HMT, Cosivi O, Schneider MC (2013). Progress towards eliminating canine rabies: Policies and
perspectives from Latin America and the Caribbean. Philos Trans R Soc Lond B Biol Sci.

[23] Matos JC, Mafra CR, Andretta AGM, Alves LR (2013). Acompanhamento antirrábico humano e consequente adesão à
profilaxia pós-exposição. Rev Enferm UFPE On Line.

[24] Santos CVB, Melo RB, Brandespim DF (2017). Perfil dos atendimentos antirrábicos humanos no agreste
pernambucano, 2010-2012. Epidemiol Serv Saude.

